# Cytotoxicity and Transcriptomic Analysis of Silver Nanoparticles in Mouse Embryonic Fibroblast Cells

**DOI:** 10.3390/ijms19113618

**Published:** 2018-11-16

**Authors:** Sangiliyandi Gurunathan, Muhammad Qasim, Chanhyeok Park, Hyunjin Yoo, Dong Yoon Choi, Hyuk Song, Chankyu Park, Jin-Hoi Kim, Kwonho Hong

**Affiliations:** Department of Stem Cell and Regenerative Biotechnology and Humanized Pig Center (SRC), Konkuk Institute of Technology, Konkuk University, Seoul 05029, Korea; gsangiliyandi@yahoo.com (S.G.); qasimattock@gmail.com (M.Q.); chanhyeok.park3751@gmail.com (C.P.); hyunjinyoo7@gmail.com (H.Y.); dongyoonchoi91@gmail.com (D.Y.C.); Songh@konkuk.ac.kr (H.S.); chankyu@konkuk.ac.kr (C.P.); jhkim541@konkuk.ac.kr (J.-H.K.)

**Keywords:** cytotoxicity, oxidative stress, antioxidants, DNA damage, epigenetics, apoptosis

## Abstract

The rapid development of nanotechnology has led to the use of silver nanoparticles (AgNPs) in biomedical applications, including antibacterial, antiviral, anti-inflammatory, and anticancer therapies. The molecular mechanism of AgNPs-induced cytotoxicity has not been studied thoroughly using a combination of cellular assays and RNA sequencing (RNA-Seq) analysis. In this study, we prepared AgNPs using myricetin, an anti-oxidant polyphenol, and studied their effects on NIH3T3 mouse embryonic fibroblasts as an in vitro model system to explore the potential biomedical applications of AgNPs. AgNPs induced loss of cell viability and cell proliferation in a dose-dependent manner, as evident by increased leakage of lactate dehydrogenase (LDH) from cells. Reactive oxygen species (ROS) were a potential source of cytotoxicity. AgNPs also incrementally increased oxidative stress and the level of malondialdehyde, depleted glutathione and superoxide dismutase, reduced mitochondrial membrane potential and adenosine triphosphate (ATP), and caused DNA damage by increasing the level of 8-hydroxy-2′-deoxyguanosine and the expressions of the *p53* and *p21* genes in NIH3T3 cells. Thus, activation of oxidative stress may be crucial for NIH3T3 cytotoxicity. Interestingly, gene ontology (GO) term analysis revealed alterations in epigenetics-related biological processes including nucleosome assembly and DNA methylation due to AgNPs exposure. This study is the first demonstration that AgNPs can alter bulk histone gene expression. Therefore, our genome-scale study suggests that the apoptosis observed in NIH3T3 cells treated with AgNPs is mediated by the repression of genes required for cell survival and the aberrant enhancement of nucleosome assembly components to induce apoptosis.

## 1. Introduction

Nanoparticles (NPs) are an emerging class of functional materials. Silver NPs (AgNPs) are widely used in various biomedical applications including antibacterial, antiviral, anti-inflammatory, and anticancer therapies. AgNPs are being used to produce various biomedical products where they are used to prevent infection [1]. Recent advances in nanotechnology have widened the potential applications for AgNPs. This has led to increased human exposure to AgNPs, which has prompted concern regarding their adverse biological effects [2,3,4]. The potential health risks associated with exposure of mammals, including humans, to AgNPs are unclear and need to be clarified. Recent studies have suggested that the toxicity due to NPs depends on the type of material and on the physical and chemical properties of the NPs. Toxicity is mainly influenced by various factors, which include cellular uptake of NPs and their subsequent interactions with cells [5,6].

AgNPs-mediated toxicity involves various mechanisms, in particular the production of excess reactive oxygen species (ROS). At low levels, ROS regulate various cellular functions and at higher levels induce cell death [7,8]. Disproportionate production of ROS induces apoptosis in a variety of human cancers, including breast cancer, ovarian cancer, and lung cancer [9,10,11]. Recent in vitro and in vivo studies have demonstrated the toxicity of AgNPs in a variety of cancerous and non-cancerous cells [9,11,12,13,14,15,16].

The conventional physical and chemical synthesis of AgNPs is simple and can result in the production of a large quantity of material. However, the process is energy-demanding and time-consuming and is not ecofriendly because of the use of harmful chemicals. Therefore, synthesis of AgNPs without the use of hazardous chemicals and the development of AgNPs with well-controlled size and tailored morphological and physicochemical features are necessary. Only a few studies have addressed the synthesis of AgNPs using a pure flavonoid reduction of Ag and its effects on the molecular mechanism of toxicity. Bio-indicators of AgNPs’ toxicity in a mammalian system would be valuable to identify. Mouse embryonic fibroblasts (MEFs) are a suitable model system to study gene ablations [17] and are also involved in wound repair and healing. AgNPs have been exploited in cancer therapy. Therefore, a comprehensive analysis of the toxicity of AgNPs effects and the molecular mechanisms of the toxicity is needed. AgNPs can modulate epigenetic dysregulation, which is involved in gene expression reprogramming, can influence the cell cycle, and can induce DNA hypermethylation following the p53 or p21 pathway, which may affect the epigenomic level [18]. 

Recent advancements in next-generation sequencing technologies have facilitated the use of high-throughput sequencing of DNA and RNA (the latter is termed RNA-Seq) from bulk or low-input samples [19]. The technology is now preferred over cDNA microarrays for the gene expression profiling of cells and tissues [20,21]. The RNA-Seq approach allows for the simultaneous analysis of a large number of genes/targets and the identification of the mechanisms of action after treatments [22,23,24,25,26,27]. However, the transcriptomic changes and cellular responses of NIH3T3 cells treated with AgNPs have not been investigated. In particular, we selected NIH3T3 as a model system for this study. Fibroblasts are ubiquitous mesenchymal cells that have important roles in wound repair and healing, and that serve as reservoirs of multipotent progenitors capable of repopulating depleted cell compartments. MEFs are frequently used to study the physiological consequences of selective gene ablations. The findings from this study using fibroblasts could provide relevant information on the potential health risks associated with human exposure to AgNPs, which should contribute to the development of safe practices for the future use of AgNPs. Understanding the mechanisms of AgNPs-mediated toxicity should also help identify avenues of protection or prevention.

This study aimed to synthesize AgNPs using myricetin. Myricetin is a flavonoid polyphenolic compound that has excellent anti-oxidant properties. We also sought to determine the effects of the synthesized AgNPs in MEFs and investigated the mechanism of action of AgNPs in regulating the growth and cellular responses of MEFs cells against AgNPs using series of cellular assays and RNA-Seq. Furthermore, gene expression profiling can be used to evaluate the molecular interactions between AgNPs and biological systems.

## 2. Results and Discussion

### 2.1. Synthesis and Characterization of Silver Nanoparticles (AgNPs) Using Myricetin

**Figure 1:**
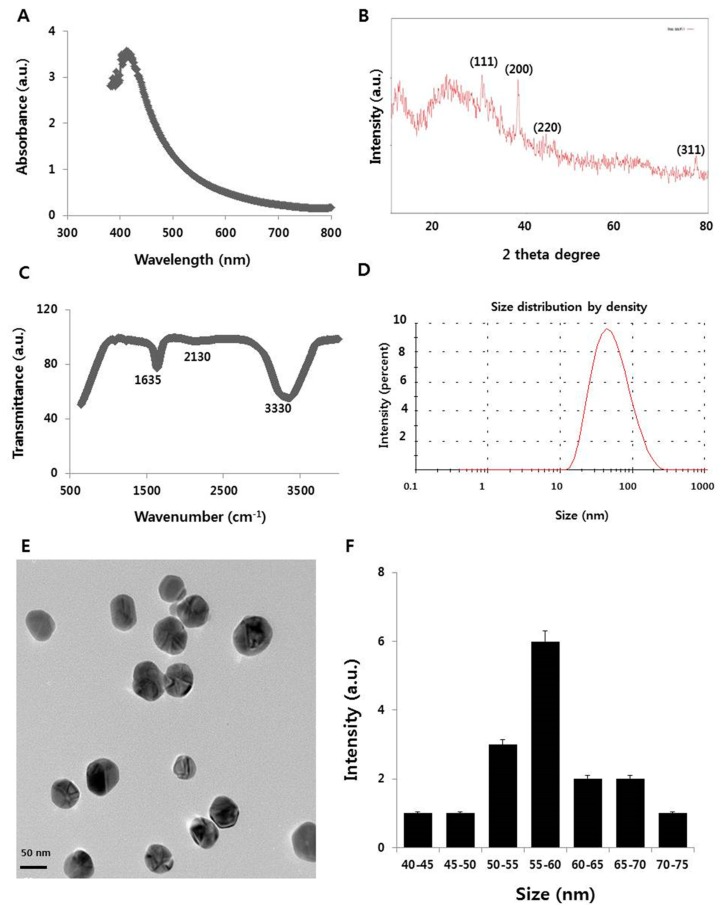

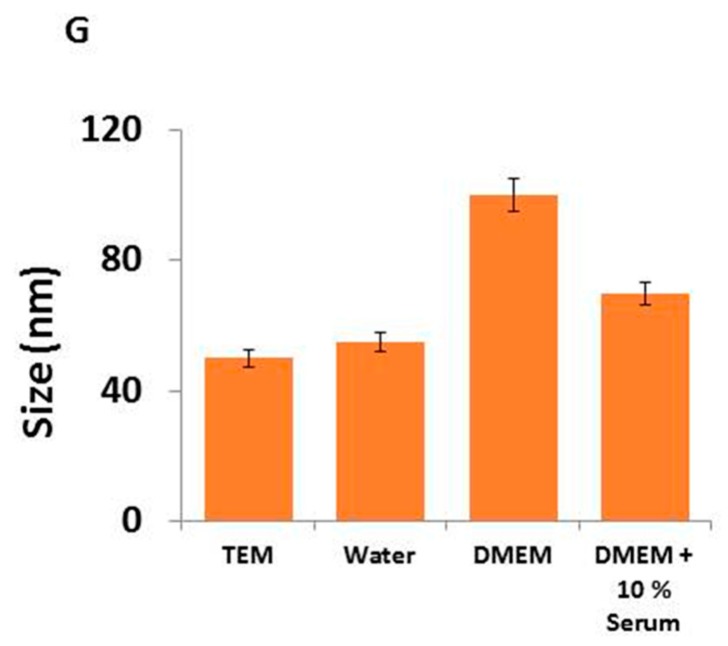
Synthesis and characterization of silver nanoparticles (AgNPs). (**A**) Ultraviolet-visible spectra of AgNPs; (**B**) X-ray diffraction pattern of AgNPs; (**C**) Fourier-transform infrared spectroscopy (FTIR) spectra of AgNP; (**D**) size distribution analysis of AgNPs using dynamic light scattering (DLS); (**E**) transmission electron microscopy (TEM) images of AgNPs; (**F**) histogram of the particle sizes from TEM images; (**G**) characterization of size distribution of AgNPs in different dispersion media.

The synthesized AgNPs was characterized by five different analytical techniques to determine the physicochemical properties of the AgNPs formed using myricetin. One was ultraviolet-visible (UV-VIS) spectroscopy (Figure 1A). A preliminary analysis provided a quick and easy screening of the synthesis of the AgNPs by identifying the localized surface plasmon resonance peak typical of nano-Ag. The Ag colloid was characterized by strong absorption in the visible region at 410 nm. The position of the maximum absorption and width of the absorption band provided information about the form, average size, and size distribution of the NPs. The average size of AgNPs was 50 ± 5 nm. As myricetin was mixed with the AgNO_3_ solution, a color change from pale yellow to colloidal brown was observed within a few hours. The obtained AgNPs emitted light between 400 and 700 nm depending on their size, shape, and morphology [28]. Flavonoids, which are present in tulsi, are able to reduce silver ions to AgNPs [29].

To identify the crystalline nature and surface morphology of the synthesized AgNPs, X-ray diffraction (XRD) analysis exhibited four different peaks (Figure 1B) at approximately 38°, 44°, 64°, and 78°, corresponding to Bragg reflection of (111), (200), (220), and (311), respectively, which are in significant agreement with a previous report [30,31]. The Debye‒Scherrer equation shows an average particle size of 55 nm.

The involvement of biomolecules in the reduction of Ag ions to AgNPs was confirmed by Fourier-transform infrared (FTIR) analysis. Figure 1C depicts the FTIR spectrum of AgNPs synthesized using myricetin as reducing agent. Peaks evident at approximately 1635, 2130, and 3330 cm^−1^ corresponded to the C=C, C≡C, and amine N‒H/O‒H vibration stretch. The results indicated that myricetin was the major biomolecule responsible for the reduction of Ag ions to AgNPs. The results are consistent with previous studies of the synthesis of AgNPs using plant extract and purified quercetin [10,29,32].

Dynamic light scattering (DLS) analysis was performed to determine the size distribution of the AgNPs synthesized using myricetin. DLS has proven to be a suitable and simple technique for the characterization of multi-modal AgNPs suspensions and is one of the most frequently used methods to obtain an average diameter of NPs dispersed in liquids. DLS is a quick, simple, and nondestructive method that can simultaneously probe many particles [33]. The DLS size distribution of myricetin-mediated synthesized AgNPs ranged from 10 to 200 nm (Figure 1D). The calculated average particle size distribution of the AgNPs was 55 nm. The broad spectrum of the DLS pattern confirmed the comparability of the particle size with the sharp SPR peak (410 nm) obtained in the UV-VIS spectra. 

To corroborate the evidence gained from the DLS size distribution, transmission electron microscopy (TEM) was done to reveal the size and morphology of the AgNPs synthesized using myricetin (Figure 1E). The prepared particles were nearly spherical in shape and uniform in size. The mean particle size obtained using myricetin as a reducing agent was 55 nm; the size significantly correlated with the absorption and XRD spectra. The TEM and DLS analyses both indicated a 55 nm particle size of the AgNPs. Sahu et al. reported that the size of NPs synthesized using hesperidin, diosmin, and naringin as the flavonoids were approximately 5–50, 5–40, and 20–80 nm, respectively [34]. Hesperidin, naringin, and diosmin-derived AgNPs were oval-shaped, polydisperse, and hexagonal in shape, respectively. Prathna et al. described an average size of 50 nm using citrus, tulsi, and quercetin plant extract produced 50, 14.6, and 11.35 nm, respectively [29,35]. Our findings suggest that myricetin produces AgNPs with an average size of 50–60 nm. These particles would easily penetrate cells and release Ag ion faster than larger particles. 

To further confirm the size distributions observed in TEM, we performed size distribution analysis in water, Dulbecco’s Modified Eagle’s Medium (DMEM) media, and DMEM with 10% fetal bovine serum (FBS) using a dynamic light scattering assay. It was found that the average size of AgNPs was 50 ± 5.0, 55 ± 6.0, 100 ± 10.0 and 70 ± 8.0 nm in TEM, water, DMEM media, and DMEM with 10% serum, respectively (Figure 1G). The results suggest that AgNPs particles dissolved in DMEM media exhibited a larger size distribution compared to AgNPs dissolved in water, whereas DMEM media with 10% FBS showed slight variation compared to DMEM alone. All these data suggest that the size distributions of AgNPs depend on the Brownian motion found in different dispersion media. Next, we analyzed the zeta potential values of AgNPs in water, DMEM, and DMEM with 10% serum, with the zeta potential values in agreement with previous observations. As shown in Table 1, absolute zeta potential values were higher when measured in DMEM or DMEM with 10% serum compared to measurements taken in media without serum.

### 2.2. AgNPs Reduce the Viability and Cell Proliferation of NIH3T3 Cells

**Figure 2:**
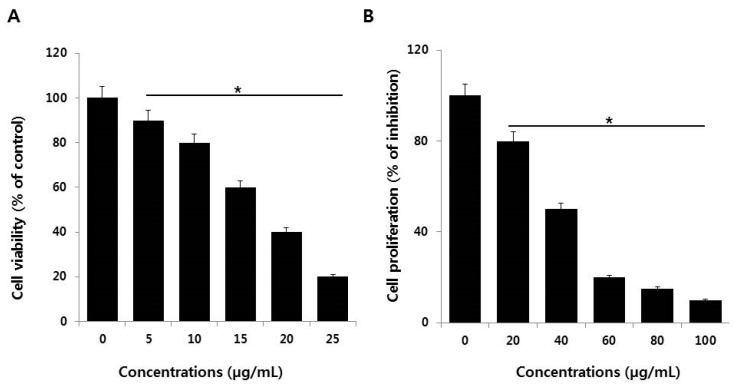
Cell viability and proliferation assessment of AgNPs in NIH3T3 cells. (**A**) Viability of NIH3T3 cells was determined 24 h after exposure to different concentrations of AgNPs using the CCK-8 assay. (**B**) Cell proliferation assay was performed using the BrdU cell proliferation assay. The results are expressed as the mean ± standard deviation of three independent experiments. There was a significant difference in the ratio for AgNP-treated cells compared to untreated cells according to a Student’s *t*-test (* *p* < 0.05).

Cell viability was determined using CCK-8. AgNPs significantly inhibited NIH3T3 viability in a dose-dependent manner (Figure 2A). Similarly, results from a cell proliferation assay showed that AgNPs significantly inhibited NIH3T3 cell proliferation, also in a dose-dependent cytotoxic manner (Figure 2B). Lee et al. [4] demonstrated that NIH3T3 cells treated for 24 h with AgNPs displayed alterations in cell morphology that included cell shrinkage, few cellular extensions, increased floating cells, accumulation of AgNPs clusters in the cytoplasm, and a dose-dependent decrease of AgNPs’ cell viability [4]. Several other studies reported that AgNPs induced toxicity by entering the cytoplasm and mitochondria through perturbations of the plasma membrane. The plasma membrane disruption increased the leakage of lactate dehydrogenase (LDH) and the generation of ROS increased cytotoxicity in a variety of human cancer cells [10,16,36]. Lee et al. [4] found that AgNPs induced ROS and heme oxidase-1 mRNA expression in NIH3T3 cells. Collectively, the data indicate the AgNPs synthesized using myricetin have dose-dependent effects on NIH3T3 cell viability and proliferation.

**Figure 3:**
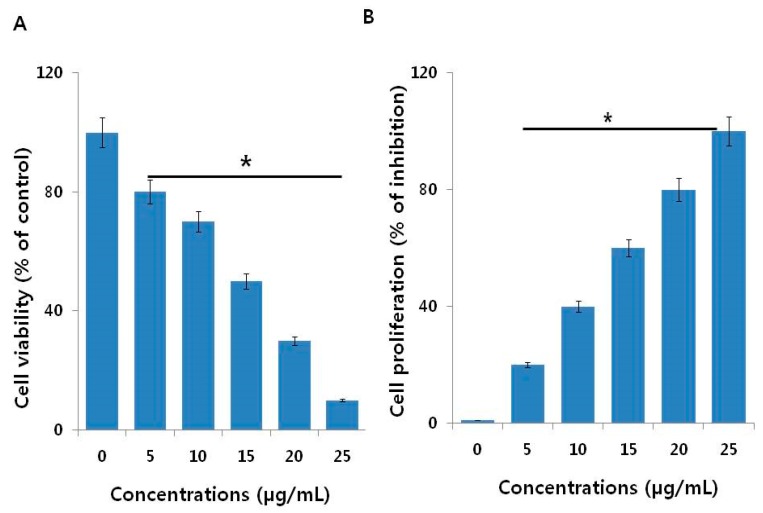
Cell viability and proliferation assessment of Ag ions in NIH3T3 cells. (**A**) Viability of NIH3T3 cells was determined 24 h after exposure to different concentrations of Ag ions using the CCK-8 assay. (**B**) Cell proliferation assay was performed using the BrdU cell proliferation assay. The results are expressed as the mean ± standard deviation of three independent experiments. There was a significant difference in the ratio of AgNP-treated cells compared to untreated cells according to a Student’s *t*-test (* *p* < 0.05).

**Figure 4:**
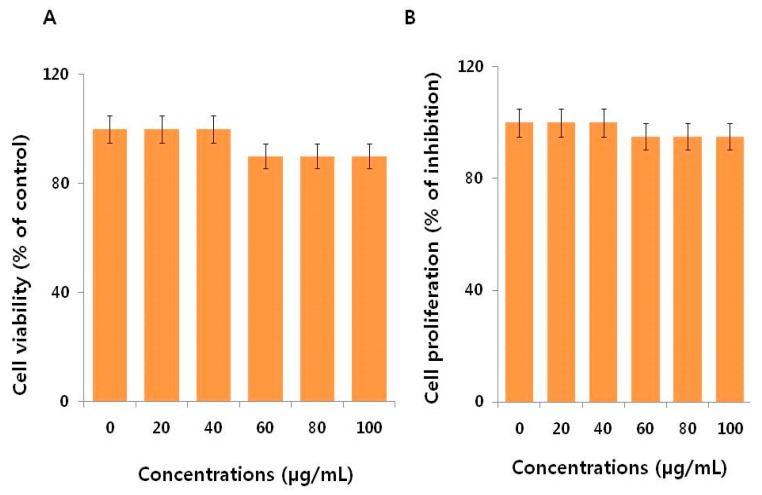
Cell viability and proliferation assessment of myricetin in NIH3T3 cells. (**A**) Viability of NIH3T3 cells was determined 24 h after exposure to different concentrations of myricetin using the CCK-8 assay. (**B**) Cell proliferation assay was performed using the BrdU cell proliferation assay. The results are expressed as the mean ± standard deviation of three independent experiments.

To determine the effectiveness of AgNPs, we performed a cell viability assay in NIH3T3 cells with various concentrations of AgNO_3_ and myricetin both used as a positive control. The viability of NIH3T3 cells decreased significantly compared to that of the negative control (Figure 3A). Notably, AgNO_3_ exhibited enhanced toxicological effects on NIH3T3 cells by decreasing cell proliferation (Figure 3B) compared to the effects of AgNPs, which is due to the fast release of silver ions from AgNO_3_ Similarly, we studied the effect of myricetin on cell viability and cell proliferation in NIH3T3 cells. The results displayed that there is no significant effect on cell viability and cell proliferation in concentrations up to 100 µg/mL (Figure 4A,B). This indicates that the concentrations of myricetin selected for the synthesis of AgNPs had no effect on cell viability and cell proliferation; the decline in cell viability and cell proliferation was merely due to AgNPs.

### 2.3. AgNPs Induce Cytotoxicity in NIH3T3 Cells

**Figure 5:**
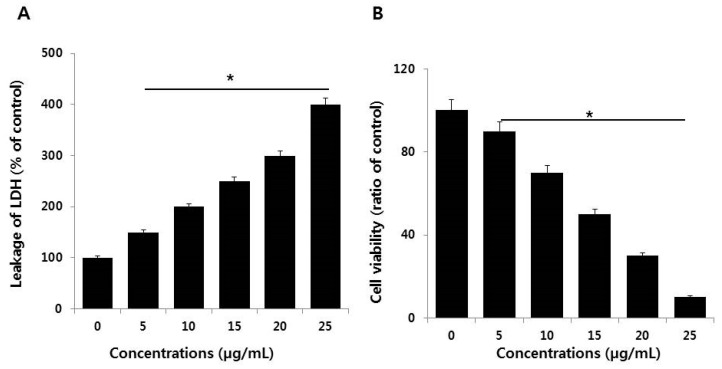
Measurement of LDH leakage and cell death protease activity in NIH3T3 cells. (**A**) LDH activity was measured at 490 nm using the LDH cytotoxicity kit. (**B**) The level of dead-cell protease was determined by the CytoTox-Glo cytotoxicity assay. The results are expressed as the mean ± standard deviation of three independent experiments. There was a significant difference in the ratio of AgNP-treated cells compared to untreated cells according to a Student’s *t*-test (* *p* < 0.05).

Cytotoxicity can be measured by the level of LDH released from cells. Normally, LDH is a cytoplasmic enzyme that is sequestered inside viable cells that have intact plasma membranes. Upon membrane damage, LDH can be released. The amount of LDH released from cells is directly proportional to the damage caused by molecules, including AgNPs. A significant effect was observed on extracellular LDH concentration even at the lowest concentration of AgNPs (5 µg/mL) (Figure 5A). This and higher concentrations produced severe leakage of LDH from NIH3T3 cells in a dose-dependent manner, suggesting that AgNPs disrupted the plasma membrane integrity of the cells, as discussed above, which is a major factor for cytotoxicity. Similarly, human and rat embryonic neural stem cells (NSCs) exposed to ≥5 µg/mL AgNPs also display significantly increased leakage of LDH [37].

Next, to corroborate the results obtained from the LDH assay, we assessed the activities of proteolytic enzymes associated with cell death or viability. The proteolytic activity is very sensitive and dependent on membrane integrity, and thus can be a good indicator of the damage caused by cytotoxic agents. To further substantiate the loss of membrane integrity, we measured cell-protease activity in NIH3T3 cells by cell viability ratio [38]. The results showed that a significant loss of viability occurred with increasing concentrations of AgNPs (Figure 5B).

### 2.4. Cellular Uptake and Internalization of AgNPs

**Figure 6:**
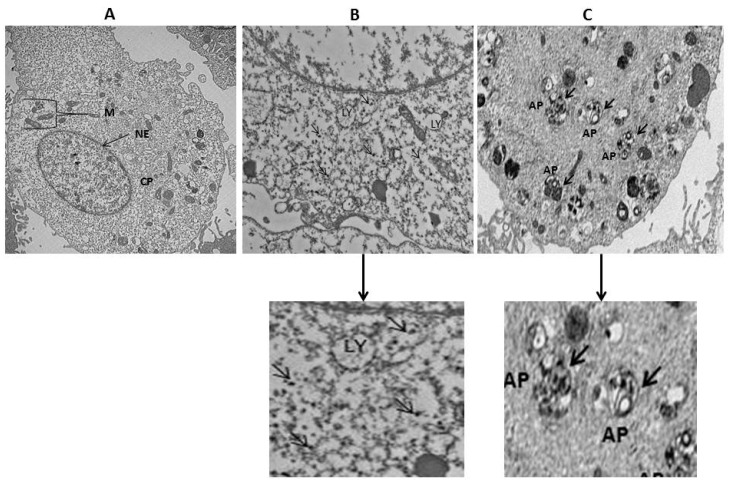
Intracellular localization of AgNPs and accumulation of autophagosomes and autolysosomes. NIH3T3 cells were treated with silver nanoparticles (AgNPs) for 24 h and then processed for transmission electron microscopy (TEM) sections. TEM images of NIH3T3 cells without AgNPs (**A**); internalization of AgNPs (thin black arrow) (**B**); and AgNPs induces accumulation of autophagosomes (thick black arrow) (**C**). M—Mitochondria; NE—Nuclear envelope; CP—Cytoplasm; LY—lysosomes; AP—Autophagosomes, scale bar: 2 µm.

To determine the cellular uptake of AgNPs by NIH3T3 cells, the cells were incubated with 15 µg/mL of AgNPs for 24 h. The TEM images of control cells showed a typical cellular structure and membranous organelles (Figure 6A), whereas micrographs of AgNPs with 15 µg/mL of AgNPs treated cells showed AgNPs in the cytoplasm (Figure 6B); in particular, AgNPs were taken up and contained mainly within membrane-bound structures (Figure 6C). Cells exposed to AgNPs exhibit typical autophagosomes (black thick arrow) with double membranes and enclosed cellular contents compared to untreated cells. In addition, when compared to the unexposed control cells, the treated cells showed many multivesicular and membrane-rich autophagosomes in close proximity to each other, indicating that AgNPs could induce autophagosome formation at the ultrastructural level [11].

### 2.5. AgNPs Induce Oxidative Stress by Increased Levels of ROS and Malondialdehyde (MDA)

**Figure 7:**
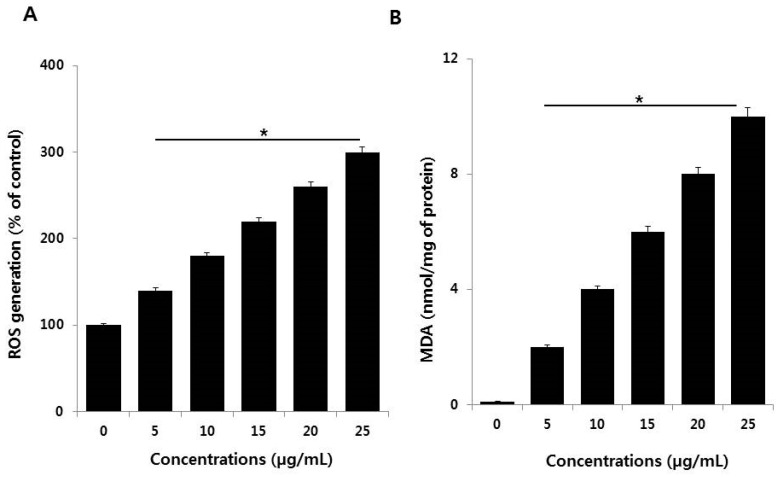
Measurement of ROS generation and malondialdehyde (MDA) in NIH3T3 cells. (**A**) NIH3T3 cells were treated with or without AgNPs for 24 h, and ROS generation was measured using DCFH-DA. (**B**) NIH3T3 cells were treated with AgNPs for 24 h. Lipid peroxidation was determined by the reaction of MDA with thiobarbituric acid to form a colorimetric (532 nm)/fluorometric (excitation and emission wavelength of 532 and 553 nm, respectively) product, whose quantity was proportional to the MDA present. The results are expressed as the mean ± standard deviation of three independent experiments. There was a significant difference in the ratio of AgNP-treated cells compared to untreated cells according to a Student’s *t*-test (* *p* < 0.05).

Of the possible mechanisms proposed for AgNPs-induced toxicity, the production of excess ROS seems to be the most important contributor for nanotoxicity or nanocytotoxicity. ROS generation is important in apoptosis induced by AgNPs. Therefore, we examined the effect of AgNPs on ROS generation in NIH3T3 cells using the 2′,7′-dichlorofluorescin diacetate (H_2_DCF-DA) assay. After a 24-h exposure, increasing concentrations of AgNPs (5–25 µg/mL) significantly increased ROS levels compared with the control (Figure 7A). Although ROS is essential for physiological activities, excess levels are potentially destructive [39,40]. The present results highlight the potential of AgNPs to induce ROS production in NIH3T3 cells. Excess ROS can injure lipids, proteins, and DNA in cells, and eventually induce apoptosis [41]. AgNPs interact with cell membrane proteins via the avid affinity of Ag for sulfur moieties. The resulting ROS production can cause the aforementioned destruction, including apoptosis and inhibition of cell proliferation [42,43,44].

To confirm the effect of ROS excess on lipid peroxidation, we analyzed the level of MDA in NIH3T3 cells treated with various concentrations of AgNPs for 24 h. AgNPs increased the level of MDA in NIH3T3 cells in a dose-dependent manner (Figure 7B). In BF-2 cells carbon NPs, such as graphene, induce increases in lipid peroxidation that are related to the concentration of graphene oxide and can become significant [45]. Other authors reported that long-term exposure of human lung cells to AgNPs produced upregulation of the expression of genes encoding anti-oxidant enzymes like glutathione-*S*-transferases, which are involved in clearing lipid peroxidation products [46]. AgNPs have the potential to induce cellular responses involved in cell survival or cell death pathways [4,47]. Several studies found that AgNPs could potentially elevate ROS generation and MDA levels in a variety of cells [13,48,49]. NIH3T3 cells displayed similar sensitivity in the AgNPs-induced loss of cell viability, cell proliferation, and cell death by increased oxidative stress and lipid peroxidation.

### 2.6. Effect of AgNPs on Antioxidants

**Figure 8:**
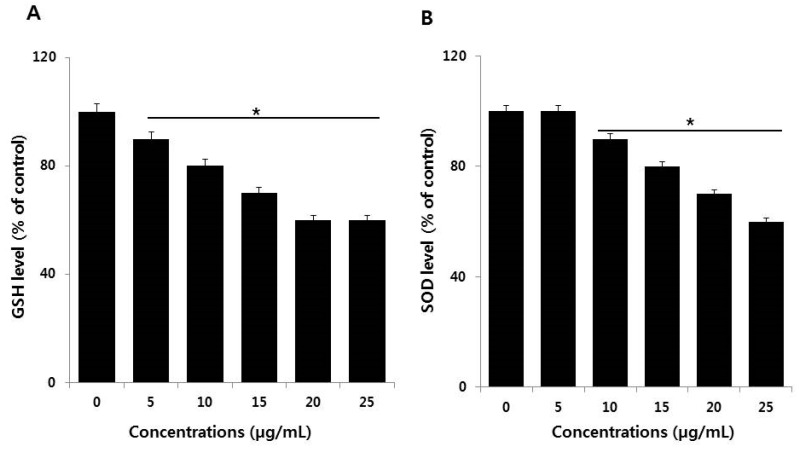
Measurement of antioxidants in NIH3T3 cells. Cells were treated with various concentrations of AgNPs for 24 h. After incubation, the cells were harvested, washed twice with ice-cold PBS, and disrupted by ultrasonication for 5 min on ice. (**A**) The concentration of glutathione (GSH) was expressed as milligram per gram of protein. (**B**) The specific activity of superoxide dismutase (SOD) was expressed as unit per milligram of protein. The results are expressed as mean ± standard deviation of three independent experiments. There was a significant difference in the ratio of AgNP-treated cells compared to untreated cells according to a Student’s *t*-test (* *p* < 0.05).

The imbalance between oxidants and antioxidants creates oxidative stress, in which the level of oxidants in cells becomes extremely high. Maintenance of the level of the reduced and oxidized (redox) state is crucial for cell viability, activation, proliferation, and organ function [50]. For example, reduced glutathione (GSH) and superoxide dismutase (SOD) are the major endogenous antioxidant scavengers. They are important in scavenging free radicals created during exposure to AgNPs. Increased oxidative stress is due to the depletion of GSH, altered level of SOD, and catalase activity in cells [51,52]. Based on this background, we examined the influence of AgNPs on the level of the GSH and SOD antioxidants. NIH3T3 cells treated with AgNPs displayed a dose-dependent decrease of GSH and SOD compared to untreated cells (Figure 8A,B). AgNPs reduced the level of GSH by increasingly inhibiting the intracellular enzyme responsible for GSH synthesis and also reduce mitochondrial membrane potentiality in buffalo rat liver (BRL) 3A rat liver [53]. Similarly, AgNPs are cytotoxic via the mitochondrial pathway by reducing GSH, enhancing lipid peroxidation, and enhancing the expression of ROS responsive genes, which cause DNA damage, apoptosis, and necrosis [44]. AgNPs were reported to cause cytotoxicity and genotoxicity through decreased mitochondrial membrane potential and reductions in the activities of SOD, catalase (CAT), glutathione peroxidase (GPx), glutathione-*S*-transferase (GST), and glutathione reductase (GR), as well as total GSH level in Chinese Hamster Ovary cells [54]. A recent study reported that the combination of palladium NPs and tubastatin-A enhances apoptosis in human breast cancer cells through reductions in the levels of various antioxidants markers, including GSH, SOD, and CAT, with oxidative stress ultimately resulting from the excess ROS [55].

### 2.7. Effect of AgNPs on Mitochondrial Dysfunction and ATP Generation

**Figure 9:**
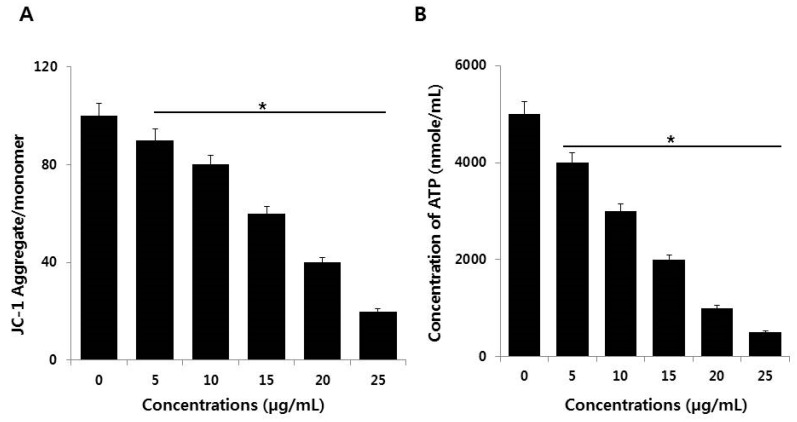
Effect of AgNPs on MMP and ATP. (**A**) NIH3T3 cells were treated with AgNPs for 24 h. The changes in mitochondrial membrane potential (MMP) were determined using the cationic fluorescent dye JC-1. (**B**) The ATP level was measured in NIH3T3 cells exposed to AgNPs for 24 h. The results are expressed as the mean ± standard deviation of three independent experiments. There was a significant difference in the ratio of AgNP-treated cells compared to untreated cells according to a Student’s *t*-test (* *p* < 0.05).

The mitochondrion is crucial in the induction of the signal for cell apoptosis. AgNPs can disrupt mitochondrial integrity, which leads to a loss of mitochondrial membrane permeability. This loss may regulate c-Jun N-terminal kinase-mediated, caspase-dependent apoptosis [53]. We assessed the effects of AgNPs on mitochondrial membrane potential (MMP) in NIH3T3 cells. MMP was determined in AgNPs-treated NIH3T3 cells (5–25 µg/mL for 24 h) using the JC-1 cationic fluorescent dye as previously described [56]. The treated cells displayed significantly altered MMP; the JC-1 aggregate/monomer ratio was decreased in AgNPs-treated cells compared to control (Figure 9A). Thus, the reductions in cell viability and proliferation and increased toxicity described in preceding sections of this paper were likely associated with mitochondrial dysfunction. AgNPs could impair mitochondrial function prior to their penetration and accumulation in the mitochondrial membrane and it can inhibit neurite outgrowth and reduces the cell survival of premature neurons and glial cells [57]. AgNPs cause cytotoxicity in Caco-2 cells through mitochondrial depolarization and depletion of antioxidants, such as GSH, and the reduction of MMP in BRL 3A rat liver cells [53,58]. AgNPs play significant role to impair mitochondrial pathway by modulating pro and anti-oxidants [44]. Our findings suggest that loss of MMP is a major mechanism of toxicity through crosstalk between mitochondria and other cell components [11,59,60].

The MMP is impacted in bioenergy disorders. It is important for ATP production and is required for biogenesis of Fe‒S clusters, to drive the mitochondrial import of many nuclear-encoded proteins, and for calcium transport [61,62,63]. We investigated whether the loss of MMP could influence the level of ATP production in AgNP-treated cells by measuring the level of ATP in AgNP-treated and untreated cells. AgNP-treated cells displayed significantly and dose-dependently reduced ATP production compared to control cells (Figure 9B). The results showed that the synthesis of ATP was compromised by AgNP treatment. The findings indicated the declining of synthesis of ATP is similar to drug-induced mitochondrial poisoning inherited mitochondrial diseases [64]. Damage to mitochondria leads to decreased level of ATP production [65]. Alteration of MMP is the potential cause for mitochondrial dysfunction, which is the major factor for the generation of ROS, lipid peroxidation, and susceptibility to apoptosis [66]. AgNPs-induced apoptosis involves the mitochondrial pathway; NIH3T3 fibroblast cells and human Chang liver cells treated with AgNPs causes release of cytochrome c release into the cytoplasm and translocation of Bax to mitochondria. Moreover, AgNPs may promote alterations in the MMP leading to stress-induced apoptotic cell death [13,16,51,53]. Thus, AgNPs could impair mitochondrial function and reduce the generation of ATP, which eventually leads to cell death.

### 2.8. AgNPs Cause DNA Damage in NIH3T3 Cells

**Figure 10:**
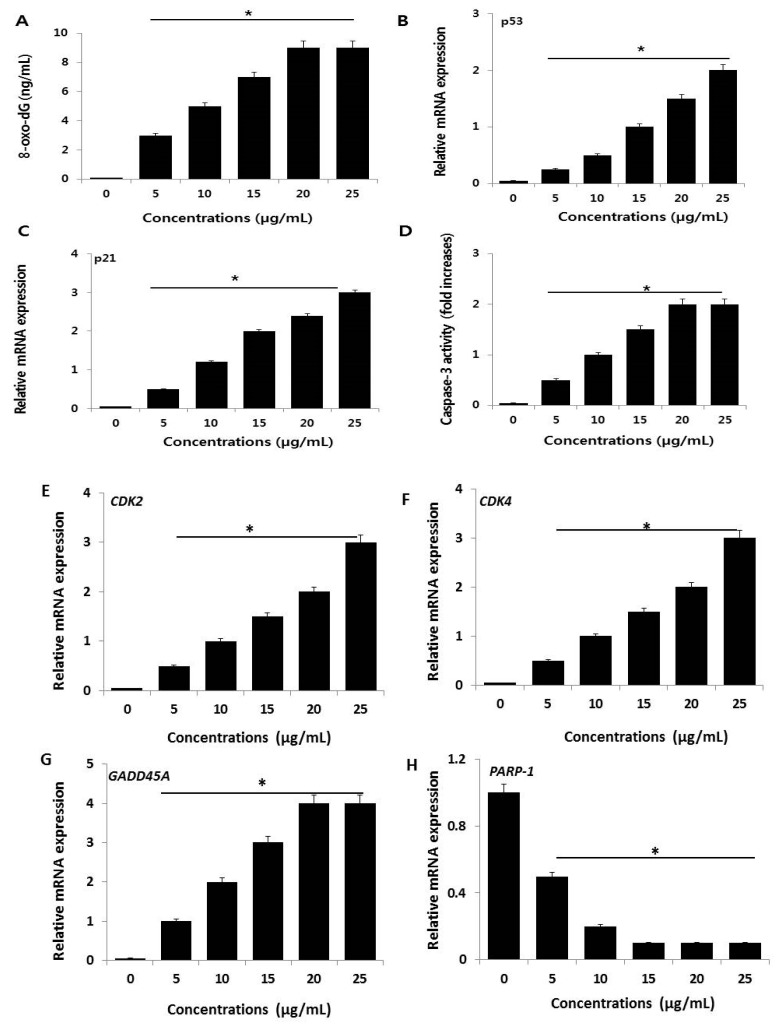
AgNPs induce DNA damage and increase expression of p53 and p21 genes in NIH3T3 cells. (**A**) 8-oxo-dG was measured after 24 h of exposure of NIH3T3 cells. AgNPs significantly increased oxidative DNA damage as evidenced by significant increases in 8-oxo-dG expressions. (**B**,**C**) Relative mRNA expression of *p53* and *p21* was analyzed using qRT-PCR in NIH3T3 cells treated with various concentrations of AgNPs for 24 h. (**D**) Caspase-3 activity was measured in AgNP-treated NIH3T3 cells. The results are expressed as the mean ± standard deviation of three separate experiments. (**E**–**H**) Relative mRNA expression of *CDK2*, *CDK4*, *GADD45A* and *PARP-1* was analyzed using qRT-PCR in NIH3T3 cells treated with various concentrations of AgNPs for 24 h. There was a significant difference in the ratio of AgNP-treated cells compared to untreated cells according to a Student’s *t*-test (* *p* < 0.05).

Excess ROS generation can permanently damage cellular membrane lipids, as well as proteins and DNA. 8-OhdG can be used to measure the effect of endogenous oxidative damage to DNA and as a factor of the initiation and promotion of carcinogenesis [67]. To evaluate the effect of AgNP-induced ROS on DNA damage in NIH3T3 cells, 8-oxo-dG levels were determined. A 24-h exposure of NIH3T3 cells to various concentrations of AgNPs increased oxidative DNA damage, as indicated by a significant elevated level of 8-oxo-dG production (Figure 10A). A prior study demonstrated that AgNPs could induce laddering of DNA and DNA fragmentation in Dalton’s lymphoma ascites cell lines [68]. Similarly, a 24-h exposure of rat NSCs to 5 µg/mL AgNPs increased the level of 8-oxo-dG. AgNP-induced oxidative stress can stimulate various genotoxic effects in variety of human cells [53,69,70]. Collectively, our findings suggest that AgNPs potentially induce oxidative damage to DNA due to an increased generation of ROS. Therefore, ROS is a major factor of AgNP-induced cytotoxicity in NIH3T3 cells. 

The mechanisms of AgNP toxicity include oxidative stress, DNA damage, and apoptosis in numerous human cell lines in vitro [53,69,70]. To further substantiate AgNP-induced DNA damage in NIH3T3 cells, we measured the expression of representative genes responsible for DNA damage (p53 and p21) in AgNP-treated and untreated cells. AgNP treatment resulted in increased expression of p53 and p21 compared to untreated cells (Figure 10B,C). These elevations might contribute to AgNPs-mediated oxidative stress, which is the major cause of DNA damage. Previous studies demonstrated that oxidative stress can induce DNA fragmentation, single-strand breaks in human breast cancer cells, and double-strand breaks in ovarian cancer cells [9,71]. Another study reported that HT22 hippocampal neuronal cells exposed to AgNPs for 24 h displayed effects on various cellular processes by alteration of p53, p21, and lamin B1, as well as methylation changes [18]. The increased level of p53 and p21 noted by us and others is responsible for DNA damage and may also account for the observed loss of cell viability and inhibition of cell proliferation. Previous report suggest that AgNPs induce cell death via activation of p53, p-Erk1/2, and caspase-3 signaling, and downregulation of Bcl-2. Our findings also confirmed that NIH3T3 cells treated with AgNPs displayed a dose-dependent increase in the expression of caspase-3 (Figure 10D). Caspase-3 is a typical mediator of cell death. AgNPs inhibit vascular endothelial factor activated cell survival and proliferation by suppression of AKT phosphorylation and induction of caspase 3 activity in bovine retinal endothelial cells [30,72]. Similarly, AgNPs cause cytotoxicity in a dose-dependent manner in cerebellum granule cells activated by the induction of caspase-3 activation, oxidative stress, reduction of antioxidants, and intracellular calcium levels [73]. Extreme level of ROS production causes the upregulation of mitogen-activated protein kinase (MAPK) P38, downregulation of total AKT, and the significantly increased expressions of caspase-3, H2A histone family member X (H2X), p-p53, and total p53 [74]. Collectively, our findings suggest that AgNPs can induce apoptosis through the activation of p53, p21, and the caspase-3-mediated signaling pathway.

To evaluate the effect of AgNPs on cell cycle arrests, the key regulators of G1/S transition, *CDK2*, *CDK4*, *GADD45A* and *PARP-1* expression levels were analyzed by RT-PCR after 24 h. *CDK2*, *CDK4*, and *GADD45A* were strongly up-regulated after AgNP treatment. *PARP-1* was strongly down-regulated after AgNP treatment (Figure 10E–H). Furthermore, decreased expression of PARP-1 resulted increased caspase-3 activity, and increased p53 and p21 gene expression in NIH3T3 cells. Overall, the expression analysis confirmed that down-expression of PARP-1 suppressed cell proliferation and induced apoptosis of NIH3T3 cells through p53 signaling pathway and also it suggest a direct effect on cell cycle regulatory proteins and confirm prevention of G1/S phase progression.

### 2.9. AgNPs Alter the Expression of Genes Involved in Apoptosis and Nucleosome Assembly

**Figure 11:**
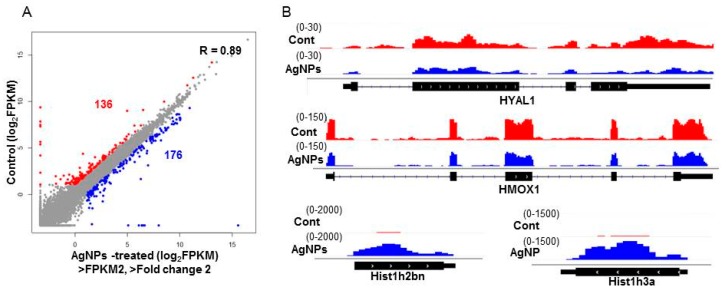
AgNPs administration changes gene expression. (**A**) A scatter plot showing genes up- or downregulated by AgNP treatment. Note that 136 and 176 genes were down- (red) and upregulated (blue), respectively. The log_2_ FPKM values were used for plotting. Cutoff value: FPKM >2 and fold change (FC) >2. (**B**) IGV tracks of representative up- (HIST1H2BN and HIST1H3A) and downregulated genes (*HYAL1* and *HMOX1*).

**Figure 12:**
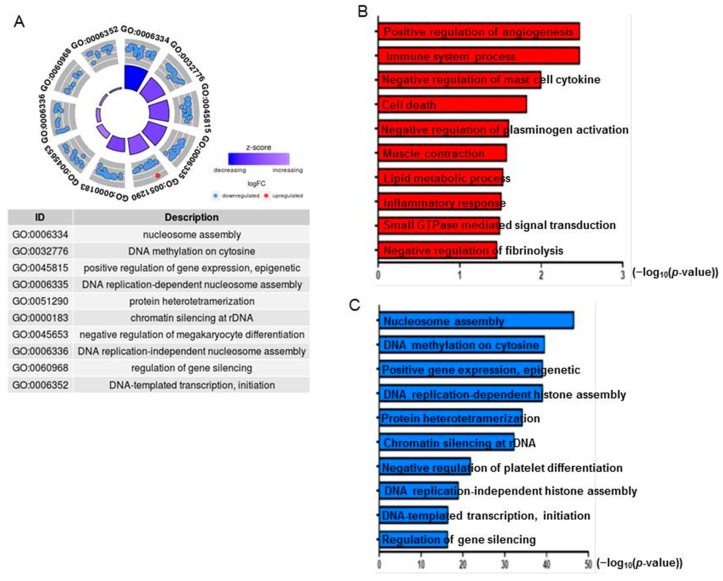
The altered gene expression is associated with apoptosis and nucleosome assembly. (**A**) gene ontology (GO) term analysis using both up- and downregulated genes together. (**B**) GO term analysis using downregulated genes only. (**C**) GO term analysis using upregulated genes only; −log_10_ (*p*-value) is indicated on the x-axis.

To investigate the mechanism by which AgNPs elicit cell apoptosis in a dose-dependent manner, RNA-Seq was performed in normal cells and cells treated with a 50% inhibitory concentration of AgNPs (12 µg/mL). We generated more than 20 million reads/sample from two biological replicas in each group. As illustrated in Figure 11A, 136 and 176 genes were down- and upregulated in AgNPs-treated cells, respectively. IGV tracks of *HYAL1*, *HMOX1*, *HIST1H2BN*, and *HIST1H3A* genes are visualized as representative genes in Figure 11B. We next investigated the biological processes impaired by AgNPs with differentially expressed genes. To that end, both up- and downregulated genes were used for gene ontology (GO) analysis. Interestingly, epigenetics-related biological processes including nucleosome assembly and DNA methylation were highly ranked in the analysis (Figure 12A). Dysregulation of epigenetics leads to cell apoptosis in normal and cancer cells [75,76]. Furthermore, accumulating evidence suggests that some NPs cause aberrant epigenetic changes, which in turn lead to detrimental effects to cell proliferation and survivor [77]. Next, we sought to determine biological processes with down- or upregulated genes. As shown in Figure 12B, positive regulation of angiogenesis and immune system process were identified as the most highly ranked biological processes with the downregulated genes. Enhanced expression of *HMOX1*, a gene associated with the positive regulation of angiogenesis, has been reported to modulate autophagy function and protect podocytes from apoptosis [78]. Also, elevated hyaluronidase 1 (HYAL1) expression affects endocytic vesicle trafficking and promotes aggressiveness of tumor cells [79]. Therefore, our RNA-Seq data suggest that the increased apoptosis of AgNPs-treated NIH3T3 cells was at least in part mediated by the repression of the genes.

**Figure 13:**
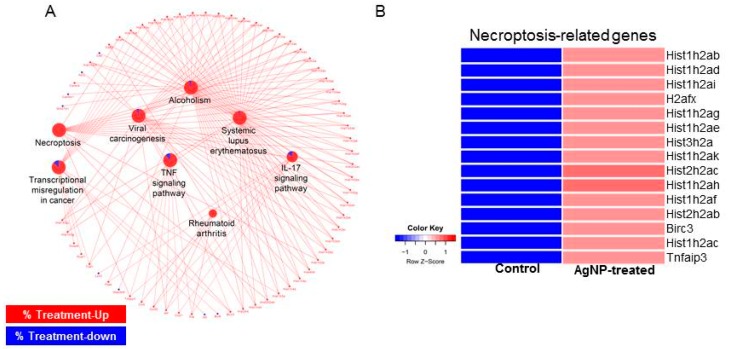
Biological pathways associated cell death are activated by AgNP treatment. (**A**) Network analysis showing aberrantly activated pathways in AgNP-treated NIH3T3 cells. Red and blue in the pie chart of pathway represent the ratio of up- and downregulated genes, respectively. (**B**) Heatmap showing expression level of genes classified in necroptosis pathway.

Using all the differentially expressed genes (DEGs), we next determined biological pathways altered by AgNP exposure. As shown in Figure 13A, pathways related to cell death such as necroptosis (necrosis + apoptosis) and tumor necrosis factor (TNF) pathways were activated by the DEGs. Many GO terms related to epigenetics were identified in the upregulated genes. In particular, expressions of histone cluster genes were increased with AgNP treatment. Previous studies reported that many different NPs and nanomaterials affect aspects of epigenetic status, such as DNA methylation and histone modification [80,81]. AgNPs can induce phosphorylation of histone H3 at serine 10 (pH3S10) by Aurora kinase in a mitosis-independent manner [82]. Consistently, a subset of histones enriched in the necroptosis pathway was shown to be enhanced in their expressions upon AgNP treatment (Figure 13B). Therefore, our RNA-Seq data suggest that the increased apoptosis of AgNP-treated NIH3T3 cells was at least in part mediated by the change of gene transcription.

Many GO terms related to epigenetics were identified in the upregulated genes. In particular, expressions of histone cluster genes were increased with AgNP treatment. Previous studies reported that many different NPs and nanomaterials affect aspects of epigenetic status, such as DNA methylation and histone modification [80,81]. AgNPs can induce phosphorylation of histone H3 at serine 10 (pH3S10) by Aurora kinase in a mitosis-independent manner [82].

To the best of our knowledge, our study is the first demonstration that NPs can alter bulk histone gene expression. How the NPs cause epigenetic changes is still unclear, as is whether their effects on gene transcription are direct or indirect. Nevertheless, our genome-scale study suggests that apoptosis observed in AgNP-treated NIH3T3 cells is mediated by the repression of genes required for cell survival and because of the aberrant enhancement of the components of nucleosome assembly.

## 3. Materials and Methods

### 3.1. Synthesis and Characterization of AgNPs

Synthesis of AgNPs was carried with myricetin dissolved in dimethyl sulfoxide (DMSO; 1 mg/mL). AgNPs were synthesized by incubating 1 mg myricetin in 100 mL of water containing 1 mM AgNO_3_ at 37 °C for 1 h [83].

### 3.2. Cell Viability and BrdU Cell Proliferation Assay

Cell viability was measured using Cell Counting Kit-8 (CCK-8; CK04-01, Dojindo Laboratories, Kumamoto, Japan). Cell proliferation was determined according to manufacturer’s instructions (Roche, Basel, Switzerland). Cells were incubated with various concentrations of AgNPs for 24 h with the BrdU labeling solution added 2 h before the end of the incubation. 

### 3.3. Membrane Integrity

The membrane integrity of NIH3T3 fibroblasts was evaluated using an LDH Cytotoxicity Detection Kit (Sigma-Aldrich, St. Louis, MO, USA). Briefly, cells were exposed to various concentrations of AgNPs for 24 h.

### 3.4. Determination of Intracellular ROS

NIH3T3 fibroblasts cells were treated with AgNPs for 24 h. ROS was measured as previously described [47] based on the intracellular peroxide-dependent oxidation of 2′,7′-dichlorodihydrofluorescein diacetate (DCFH-DA; Molecular Probes, Eugene, OR, USA) to form the fluorescent compound 2′,7′-dichlorofluorescein (DCF). 

### 3.5. Measurement of Oxidative and Antioxidant Markers

The oxidative stress markers MDA, GSH, and SOD were assayed according to the manufacturer’s instructions (Sigma-Aldrich, St. Louis, MO, USA).

### 3.6. JC-1 Assay and Measurement of ATP

NIH3T3 fibroblasts were treated with AgNPs for 24 h. The change in MMP was determined using the cationic fluorescent dye JC-1 (Molecular Probes). The ATP level was measured in NIH3T3 cells according to the manufacturer’s instructions (Catalog Number MAK135, Sigma-Aldrich). The cells were exposed to various concentrations of AgNPs for 24 h and the level of ATP was measured.

### 3.7. Measurement of 8-oxo-dG

8-oxo-dG was determined as described previously [37] and also according to manufacturer’s instructions (Trevigen, Gaithersburg, MD, USA).

### 3.8. Measurement of Caspase 3 Activity

The caspase-3 activity was measured according to manufacturer instructions. The cells were treated with AgNPs for 24 h, and then the activity of caspase-3/9 was measured in the cancer cells using a kit from (Sigma-Aldrich, St. Louis, MO, USA). The calorimetric assay was based on the hydrolysis of the caspase-3 substrate by caspase-3, resulting in the release of the p-nitroaniline (pNA) moiety. The concentration of pNA released from the substrate was calculated from the absorbance values at 405 nm. 

### 3.9. Reverse Transcription-Quantitative Polymerase Chain Reaction (RT-qPCR)

Total RNA was extracted from the cells treated with various concentrations of AgNPs for 24  h using the PicoPure RNA isolation kit (Arcturus Bioscience, Mountain View, CA, USA). Samples were prepared according to the manufacturer’s instructions. Real-time RT-qPCR was conducted using a Vill7 (Applied Biosystems, Foster City, CA, USA) and SYBR Green as the double-stranded DNA-specific fluorescent dye (Applied Biosystems). Target gene expression levels were normalized to the expression of *glyceraldehyde-3-phosphate dehydrogenase* (*GAPDH*) expression, which was unaffected by treatment. The real-time qRT-PCR primer sets are shown in Table 2. 

### 3.10. RNA-Seq and Downstream Bioinformatics Analysis

Total RNA was obtained from control and AgNs-treated cells using an RNeasy kit (Qiagen, Valencia, CA, USA), and their concentration and quality for library preparation was determined using Bioanalyzer RNA chip (Agilent Technologies, Santa Clara, CA, USA). RNAs with an RNA integration number >7 were used for library preparation using TruSeq stranded total RNA sample preparation kit (Illumina, San Diego, CA, USA). Briefly, the total RNAs were subjected to cDNA synthesis, fragmentation, adaptor ligation, and PCR amplification. Sequencing was performed on the NextSeq500 platform (Illumina). After sequencing, the sequencing reads were further processed with determination of quality and trimming the adaptors using the FastQC tool. The clean reads were mapped to mouse mm9 genome using the STAR package. The fragments per kilobase million (FPKM) and differentially expressed genes (DEGs) were obtained using the following Cufflink (version 2.2.1, Seattle, WA, USA) option: -library-type = fr-second strand. DEGs was determined at FPKM ˃2 and fold change (FC) ˃2. DAVID (version 6.8, Frederick, MD, USA) tool was used to determine enrichment of biological processes. R package (version 3.3.2, Vienna, Austria) and GO plot package (version 1.0.2, Madrid, Spain) were used for generation of scatter plot and GO terms, respectively. Biological pathways were determined using ClueGO tool plugged in Cytoscape (version 3.6.1, La Jolla, CA, USA).

### 3.11. Statistical Analyses

All assays were conducted in triplicate, and each experiment was repeated at least three times. The results represent the means of at least three independent experiments (mean ± standard deviation). Student’s *t*-test or one-way analysis of variance, followed by Tukey’s test for multiple comparisons were calculated, using GraphPad Prism software (GraphPad Software, San Diego, CA, USA). Differences were considered significant at *p* < 0.05. 

## 4. Conclusions

The increasing use of AgNPs in consumer, industrial, and biomedical products has prompted global concern regarding their toxicity and their impact to biological systems. We prepared AgNPs using the flavonoid myricetin. The synthesized AgNPs were characterized by various analytical techniques. The prepared AgNPs had an average size of 55 nm and were uniformly spherical in shape. Next, we examined the effects of biogenic AgNPs on NIH3T3 cells to decipher the molecular mechanism of nanotoxicity and biochemical pathways involved. AgNPs induced the loss of cell viability and proliferation via increased leakage of LDH, the generation of ROS, increased MDA levels and decreased levels of antioxidants. AgNPs caused mitochondrial dysfunction through alteration of membrane permeability and generation of ATP. The induction of apoptosis by DNA damage was apparent by the increased level of 8-oxodG and increased expressions of p53 and p21. The AgNP-induced death of NIH3T3 cells suggests that NP-induced oxidative stress could have an important influence on NIH3T3 cell survival. AgNP-induced oxidative stress can eventually cause genotoxicity by DNA adducts, DNA breaks, and increased 8-oxoguanine level. RNA-Seq analysis suggested that AgNPs alter epigenetics-related biological processes including nucleosome assembly and DNA methylation. AgNPs could also influence the cell cycle, and induce DNA hypermethylation via the p53 or p21 pathway, which may have an effect on epigenomic level. Our genome-scale study suggested that the apoptosis observed in AgNPs-treated NIH3T3 cells is mediated by the repression of genes required for cell survival and by the aberrant enhancement of components of nucleosome assembly, with the resulting induction of apoptosis. This combination study of cellular assays and RNA-Seq is the first report demonstrating that AgNPs can alter cellular responses and bulk histone gene expression in MEFs, which provides an in vitro model system to study various biological events as well as disease states. 

## Figures and Tables

**Table 1:** Zeta potential of AgNPs in different dispersion media.

Solvent	Zeta Potential
Water	−25.2 ± 0.1
DMEM	20.5 ± 1.5
DMEM + 10% serum	11.5 ± 2.2

**Table 2:** List of primers used for quantitative real-time polymerase chain reaction for analysis of apoptotic and cell cycle arrest gene expression.

Gene	Primer
**p53**	**F:** AGAGACCGTACAGAAGA
	**R:** CTGTAGCATGGGATCCTTT
**p21**	**F:** GTTGCTGTCCGGACTACCG
	**R:** AAAAACAATGCCACCACTCC
**PARP-1**	**F:** CTCCATCCTGGCCTCGCTGT
	**R:** GCTGTCACCTT CACCGTTCC
**CDK2**	**F:** GCTAGCAGACTTTGGACTAGCCAG
	**R:** AGCTCGGTACCACAGGGTCA
**CDK4**	**F:** CTGGTGTTTGAGCATGTAGACC
	**R:** AAACTGGCGCATCAGATCCTT
**GAPP45A**	**F:** TGCTCAGCAAAGCCCTGAGT
	**R:** GCTTGGCCGCTTCGTACA
**GAPDH**	**F:** TGCACCACCAACTGCTTAGC
	**R:** GGCATGGACTGTGGTCATGAG
